# Do the evolutionary interactions between moths and bats promote niche partitioning between bats and birds?

**DOI:** 10.1002/ece3.8355

**Published:** 2021-11-19

**Authors:** Lorinda S. Bullington, Mathew T. Seidensticker, Nathan Schwab, Philip W. Ramsey, Kate Stone

**Affiliations:** ^1^ MPG Ranch Missoula Missoula Montana USA; ^2^ Department of Ecosystem and Conservation Sciences University of Montana Missoula Montana USA; ^3^ Northern Rockies Research & Educational Services Lolo Montana USA; ^4^ Tetra Tech Portland Oregon USA

**Keywords:** bats, birds, crane flies, diet metabarcoding, diet partitioning, DNA metabarcoding, insectivore, nightjars, tympanal moths

## Abstract

Ecological theory suggests that the coexistence of species is promoted by the partitioning of available resources, as in dietary niche partitioning where predators partition prey. Yet, the mechanisms underlying dietary niche partitioning are not always clear. We used fecal DNA metabarcoding to investigate the diets of seven nocturnal insectivorous bird and bat species. Low diet overlap (2%–22%) supported resource partitioning among all species. Differences in diet corresponded with species identity, prey detection method, and foraging behavior of predators. Insects with ultrasonic hearing capabilities were consumed significantly more often by birds than bats, consistent with an evolved avoidance of echolocating strategies. In turn, bats consumed a greater proportion of noneared insects such as spruce budworms. Overall, our results suggest that evolutionary interactions among bats and moths translate to dietary niche partitioning and coexistence among bats and nocturnal birds.

## Graphical abstract

Eared moths avoid predation by echolocating bats, only to be eaten by nocturnal birds with silent flight capabilities. This suggests that the evolutionary arms race between moths and bats extends to resource partitioning among bats and birds.

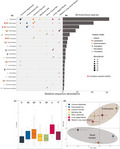

## 1 INTRODUCTION

Aerial insectivores like birds and bats are decreasing at alarming rates across North America (Spiller & Dettmers, 2019), in part due to simultaneous declines of aerial insects (Sánchez‐Bayo & Wyckhuys, 2019). Niche theory predicts that in resource‐limited environments, species that occupy the same guild will partition dietary resources to avoid competitive exclusion (MacArthur & Levins, 1964). Such partitioning is often underpinned by variations in morphology or behavior that allow species to exploit different resources (Schoener, 1974). Dietary niche partitioning related to prey size (Vesterinen et al., 2018), predator morphology, and echolocation behavior (Emrich et al., 2014) is evident among many sympatric bat species. If and how dietary partitioning occurs among co‐occurring nocturnal insectivorous birds and bats is less clear, but by identifying the processes that promote the coexistence of aerial insectivores, we can better predict future community dynamics.

Interactions between bats and moths provide a model system for studying the evolution of predator‐prey relationships (Hofstede & Ratcliffe, 2016; Waters, 2003). Prey capture by bats is often dependent on echolocation behavior and how insects respond (Fenton & Fullard, 1979). Moths with ultrasound‐sensitive ears can hear echolocation calls at distances up to 100 m (e.g., noctuids; Miller & Surlykke, 2001) and avoid predation through evasive maneuvers or sounds (Dunning et al., 1996). This adaptation arose independently in moths at least six times (Hofstede & Ratcliffe, 2016). In turn, some bats echolocate at low intensities or high enough frequencies to go undetected by moths (Faure et al., 1990; Hofstede & Ratcliffe, 2016). Yet, how evolutionary interactions between moths and bats may extend to dietary resource partitioning between bats and nocturnal insectivorous birds is unknown (Yack et al., 2020). Nocturnal birds often use visual cues and possess adaptions for silent flight that enable them to evade detection by insects (Clark et al., 2020). These adaptations may allow them to exploit resources that bats cannot. For example, eared moths can only detect the cyclic wingbeats of approaching birds within 2.5 m (Fournier et al., 2013), perhaps making moths more vulnerable to predation by visually‐oriented insectivores. The distributions of bats and nocturnal insectivorous birds suggest that they may interact. However, little research exists on if, or to what extent they may partition prey resources, or the underlying mechanisms (Fenton & Fleming, 1976).

We used fecal DNA metabarcoding to analyze the diets of seven co‐occurring nocturnal aerial insectivores (hereafter NAIs). We compared the diet composition and richness of three nocturnal birds: *Chordeiles minor* (Common Nighthawks), *Phalaenoptilus nuttallii* (Common Poorwills), *Psiloscops flammeolus* (Flammulated Owls), and four bat species: *Eptesicus fuscus* (Big Brown Bats), *Lasionycteris noctivagans* (Silver‐haired Bats), *Myotis Volans* (Long‐legged Myotis), and *Myotis evotis* (Western Long‐eared Myotis). Despite differences in prey detection methods used by these insectivores (Table 1), previous studies using microscopy of fecal samples have reported broad similarities in the insects they consume, primarily moths and beetles (Agosta, 2002; Csada et al., 1992; Ober & Hayes, 2008; Reynolds & Linkhart, 1987; Todd et al., 1998; Whitaker, 1995). However, NAI diets and available prey can vary across regions and over time, hampering cross‐study comparisons. Additionally, traditional methods of prey analysis in feces primarily result in prey identification to only the order or family level, which masks resource partitioning at finer taxonomic resolutions.

**TABLE 1:** Characteristics of nocturnal insectivores included in diet analyses

	Number of samples	Average weight of local captures (g)	Prey detection method	Foraging behavior	Diet turnover
Common Nighthawks	17	81.6 (60–105)	Visual	Open aerial hawking	0.31 ± 0.002
Flammulated Owls	16	58.3 (51.0–78.5)	Visual	Sit‐and‐wait (sallys from perch, gleans from ground, trees, or shrubs)	0.59 ± 0.004
Common Poorwills	73	49.3 (34.5–74.0)	Visual	Sit‐and‐wait (sallys from ground)	0.20 ± 0.001
Big Brown Bats	27	18.9 (13.4–30)	Echolocation	Open aerial hawking	0.40 ± 0.002
Silver‐haired Bats	26	13.4 (10.6–19.2)	Echolocation	Open aerial hawking	0.32 ± 0.002
Long‐legged Myotis	19	7.9 (5.1–11.8)	Echolocation	Open aerial hawking	0.46 ± 0.004
Western long‐eared Myotis	17	6.4 (4.8–8.6)	Echolocation and hearing	Open aerial hawking/ gleaning from trees or ground	0.43 ± 0.003

Note

Sample number, average mass of local specimens, prey detection methods, foraging behavior, and diet turnover (diet variation among individuals) of the seven nocturnal aerial insectivores sampled for dietary analysis.

As with differences in prey detection methods, NAIs in this study also display different foraging behaviors. For example, Flammulated Owls (Goggans, 1985) and Common Poorwills (Brigham & Barclay, 1992) are sit‐and‐wait predators (Table 1). Both use their legs to launch after prey from the ground or perches, a foraging behavior not found in insectivorous bats. Modifications of the pelvis that allow bats to hang from perches and fly prevent bats from jumping into flight (Schutt et al., 1997). Instead, the bats in this study hunt by foraging insects while in flight, termed “aerial hawking” (Saunders & Barclay, 1992), or, as in Long‐eared Myotis, sometimes also by gleaning insects from the ground and foliage (Faure & Barclay, 1994). Like bats, Common Nighthawks are also aerial hawkers and prey on insects at a wide range of heights above ground and over great distances in a single foraging bout (Clark et al., 2020).

Despite clear differences in prey detection and foraging behavior of insectivores, it is not always clear if or to what extent these differences translate to differences in diet. Insectivores with different foraging behaviors may still target the same prey (Brigham & Fenton, 1991; Kent & Sherry, 2020). Prey movement may also overlap with the foraging range of more than one predator species (Remmel et al., 2011). Still, foraging behaviors and prey detection methods that do correspond to dietary differences may decrease interspecific competition among NAIs.

To our knowledge, this is the first study to use fecal DNA metabarcoding to investigate the diets of multiple, distantly related, co‐occurring NAIs. Our objectives were two‐fold. First, we developed a reference barcode database from 56,191 locally collected arthropod specimens to provide more accurate taxonomic assignments of potential prey items than possible in previous studies. We then used DNA metabarcoding of fecal samples to determine the degree to which NAI diets differ in richness and composition. We expected that differences in diet would depend on NAI species identity and correspond with (1) prey detection methods (i.e., echolocation or visual hunting) and (2) differences in foraging behavior (i.e., aerial hawking or sit‐and‐wait predators).

## 2 MATERIALS AND METHODS

### 2.1 Study area

The study area encompassed c. 3500 hectares of conservation property in western Montana (www.mpgranch.com; 46°41′N, 114°00′W). Historic management practices include cattle grazing, logging, and agriculture. Current conservation strategies include restoring native grasslands and shrublands, primarily through weed control, seeding and planting efforts, wildlife management, and irrigation. Sampling occurred in mid‐elevation forest/grassland, mid‐elevation forest, floodplain forest, mid‐elevation sagebrush, mid‐elevation sagebrush/woodland, and mid‐elevation shrubland/grassland plant communities. Elevation ranged from approximately 970 m in floodplain areas to around 1650 m in higher elevation forests.

### 2.2 Sample collection and processing

We collected fecal samples from NAIs May through September during 2017 and 2018. We captured bats monthly after evening emergence in mist nets set over dry land, streams, and ponds. We placed bats in individual paper bags to collect their fecal pellets. Six additional bat species occur in the study area but were excluded from this study due to low sample sizes. We collected fresh fecal samples from Flammulated Owls, Common Poorwills, and Common Nighthawks captured in mist nets on or near breeding territories. Common Nighthawks and Common Poorwills were also sampled opportunistically near roads or at known nest or roost sites via hand nets. We placed all fecal samples in vials containing ethanol in the field and stored them in a freezer at −20°C until further processing. We labeled samples by the plant community in which they were collected, though sampling location does not always equate to plant community used while foraging. A subsample of Common Nighthawks, Flammulated Owls, and Common Poorwills was also fitted with GPS tracking devices to gather data on home and foraging ranges. Telemetry data for Common Nighthawks indicated foraging ranges up to 400 ha, whereas Common Poorwills ranged between 0.5 and 3.0 ha and Flammulated Owls generally foraged in <1.0 ha (Table A1 in Appendix 1). The daily foraging range of bats sampled varies between <1 km for Long‐eared Myotis to >4.4 km for Big Brown Bats (Maxell, 2015). Based on observational and telemetry data, home and foraging ranges overlapped for all species.

The Canadian Centre for DNA Barcoding (CCDB) performed all DNA extractions, amplification, and sequencing. DNA extraction and PCR amplification followed CCDB protocols as described in Moran et al. (2019). Samples were incubated overnight in a lysis buffer, concentrated by centrifugation, dried, and finally eluted using a Tris‐HCl elution buffer. The CCDB also processed negative extraction and PCR controls in parallel with samples. All negative controls ensured that contamination did not occur. The cytochrome C oxidase 1 (CO1) region was amplified from each sample using the arthropod‐specific primers, ZBJ‐ArtF1c_t1 and ZBJ‐ArtR2_t1 (Zeale et al., 2011), as described previously (Moran et al., 2019; Prosser & Hebert, 2017). Following amplification, samples were pooled and purified. The CCDB performed sequencing on an Ion Torrent PGM following standard protocols (Prosser & Hebert, 2017).

### 2.3 Constructing a DNA barcode library from local Arthropoda

**FIGURE A1:**
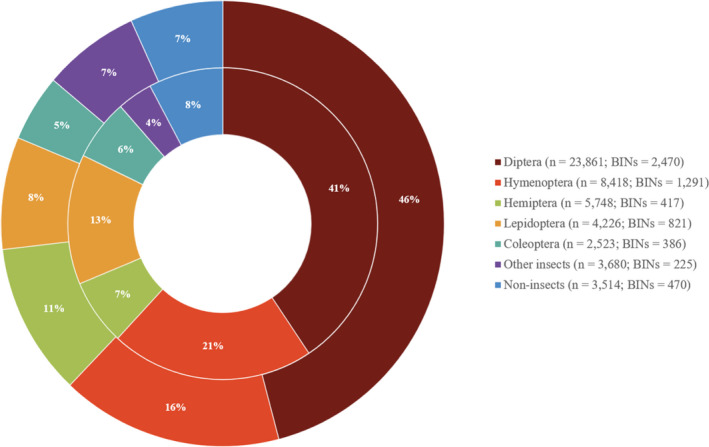
Distribution of insects in trap collections. Composition of the DNA barcode library compiled from trap collections in the study area, shown as the percentage of collected specimens assigned to each arthropod order (outer ring) and the percentage of total BINs (richness) represented by each arthropod order (inner ring). Orders representing <1% of specimens or BINS mainly included Dermaptera (earwigs), Raphidioptera (snakeflies), Trichoptera (caddisflies), Neuroptera (lacewings, mantidflies, antlions), Ephemeroptera (mayflies), and Plecoptera (stoneflies)

In 2017 and 2018, we collected nocturnal insects monthly May–August using mercury vapor and black lights placed in front of a white sheet and an aerial flight‐intercept trap at sites across our study area. In 2019, we expanded insect sampling to include bulk samples collected weekly over 13 weeks (May‐August) from flight‐intercept, pitfall, and yellow and blue pan traps. We sent samples to the CCDB for sequencing and identification (deWaard et al., 2019; Ratnasingham & Hebert, 2007). Technicians at the CCDB counted total insect abundance by order, weighed biomass, and collected tissue samples from several representatives of each morphospecies. All records are publicly available on Barcode of Life Database (BOLD) under the datasets MPG and MPGR with photos of specimens to aid in future identification. The resulting local arthropod DNA barcode library consisted of 56,191 Arthropoda specimens collected May–September from 2017 to 2019 at 48 sites within our study area. Nearly all (99.5%) of the specimen sequences were assigned to order, 92.8% to family, 58.0% to genus, and 24.4% to species. A total of 52,033 of the sequences gained Barcode Index Numbers (BINs) in the BOLD, comprising 6080 total unique BINs. This effort added 1529 previously undocumented arthropod records to BOLD, and represented 38 orders, 383 families, 1810 genera, and 1740 total species. Dominant orders represented in the final database included Diptera, Hymenoptera, Hemiptera, Lepidoptera, and Coleoptera (Figure A1).

### 2.4 Data analysis

We processed demultiplexed sequences using QIIME2 version 2020.2 (Bolyen et al., 2018). We removed all primers prior to analysis using the cutadapt plugin (Martin, 2011) and denoised sequences using the DADA2 denoise‐pyro plugin (Callahan et al., 2016). DADA2 is sensitive to single base‐pair differences among sequences and produces unique “amplicon sequence variants” (ASVs). The median base pair quality score for all sequences was maintained above 25. Denoised sequences shorter than 100 bp were removed from analyses. This resulted in a total of 1,450,971 quality‐filtered sequences. We then clustered sequences into operational taxonomic units (OTUs) based on a 97% sequence similarity threshold (Vamos et al., 2017), using the VSEARCH plugin (Rognes et al., 2016). We removed sequences only occurring in a single sample or that were represented by fewer than 0.001% of sequences to limit artifactual sequences.

**FIGURE A2:**
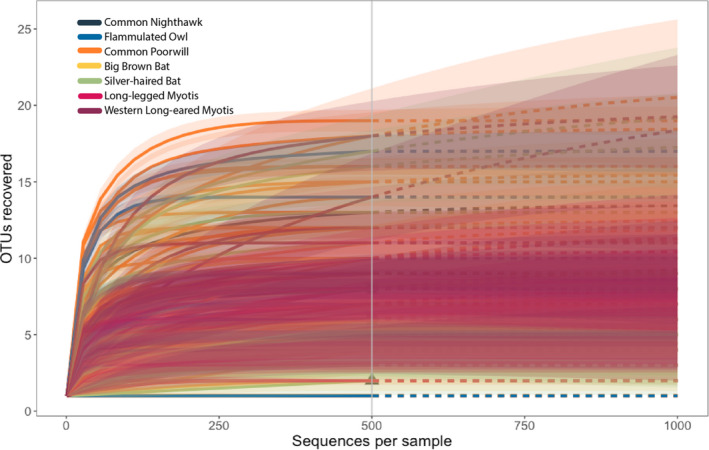
Sequencing rarefaction curve. Sequence rarefaction curve showing the number of OTUs recovered as a result of sequencing depth per sample. Grey vertical line represents sequence rarefaction depth

We determined taxonomic assignments using our local DNA barcode library and the BLAST plugin within QIIME2, with a coverage value of 0.7 and sequential percent matching identities of 100%, 99%, 98%, and 97%. If taxonomy could not be assigned to our local database using these parameters, we used a global COI database compiled from BOLD and GenBank and a pretrained RDP classifier (Porter & Hajibabaei, 2018; Wang et al., 2007). The BOLD accession ID associated with each taxonomic identification is indicated where available. We verified all taxonomic identifications based on the plausibility that they may occur within or nearby the study area. All sequences not matching to Arthropoda using either the global COI database or the local database were removed from further analyses, resulting in a total of 1,147,127 sequences with assigned taxonomy. We rarefied samples at 500 sequences per sample, which was sufficient to adequately characterize most species within each sample (Figure A2). In total, 77% of OTUs recovered from NAI fecal samples matched 97% or greater with locally collected specimens, while 23% were assigned taxonomy using the RDP classifier.

All statistical analyses were conducted in RStudio Version 1.1.453 using R version 3.6.0, (R Core Team, 2018). To identify prey taxa maximally associated (*p* < .05) with NAI species, detection methods, or foraging behaviors, we conducted multipattern analyses using the “multipatt” function in the indicspecies package (De Cáceres & Legendre, 2009) with 9999 permutations, and all *p*‐values were adjusted for multiple comparisons (Benjamini & Hochberg, 1995). Insect taxa <200 reads were removed prior to analysis.

To determine resource partitioning among NAI diet composition, we analyzed both relative read abundance and presence/absence data. Presence/absence data are considered a more conservative option in insectivore fecal analyses (Jusino et al., 2019). However, presence/absence data can also overestimate the importance of prey consumed in small quantities, and it is generally thought that relative read abundances provide more accurate population‐level data (Deagle et al., 2019). Even so, we chose to analyze both relative read abundance and presence/absence data and found similar results. We performed all compositional comparisons on either Bray‐Curtis distances of Hellinger transformed relative read abundances, or Raup‐Crick transformed presence/absence data using the vegan package (Oksanen et al., 2019).

Because differences in diet composition can stem from differences among group centroids or group dispersions, we tested for both at the OTU level. We assessed differences in diet dispersion (distance from mean) among species, prey detection methods, and foraging behaviors using the betadisper() function in the vegan package (Oksanen et al., 2019). We observed no differences in data dispersion among species or groups of species (*p* > .1).

A perMANOVA analysis was performed to test the effects of prey detection method, foraging behavior, species identity, sampling month, plant community, the presence of a water body at the sampling site, and all interactions, on abundance and presence/absence data using the adonis2 function, with permutations constrained within collection year. We applied forward selection to successively add predictor variables that significantly (*p* < .05) improved model fit. To additionally test for differences in diet for each species pair, we ran pairwise analyses using the “pairwiseAdonis2” function in the pairwiseAdonis package (Arbizu, 2021), and adjusted for multiple comparisons (Benjamini & Hochberg, 1995). We performed a principal coordinate analysis (PCoA) using the “cmdscale” function to visualize diet variation among species. For each NAI, we also calculated diet turnover among samples using the “turnover” function in the vegetarian package (Charney & Record, 2012). Diet turnover within each NAI species was calculated based on Shannon beta diversity where zero equals no difference between samples and one represents completely different samples. Standard error was estimated for diet turnover through bootstrapping, with 500 iterations. Overlap in diet was calculated based on the proportion of OTUs common to each species pair.

To accommodate non‐normal error distributions associated with richness and diversity metrics, we used generalized linear regression using the “glm” function with Gaussian or Poisson distributions to assess variation based on species, plant community, and collection month. The Akaike information criterion was used to select the best models. We used a two‐way Anova (car package, Fox & Weisberg, 2019) with a type II sum of squares for unbalanced data to test significance of predictor variables (Table A8 in Appendix 1). Where significant, the “emmeans” function in the emmeans package was used for pairwise analyses of diversity metrics between each species (Lenth et al., 2021).

## 3 RESULTS

### 3.1 Dietary partitioning corresponds with predation of eared insects

**FIGURE 1:**
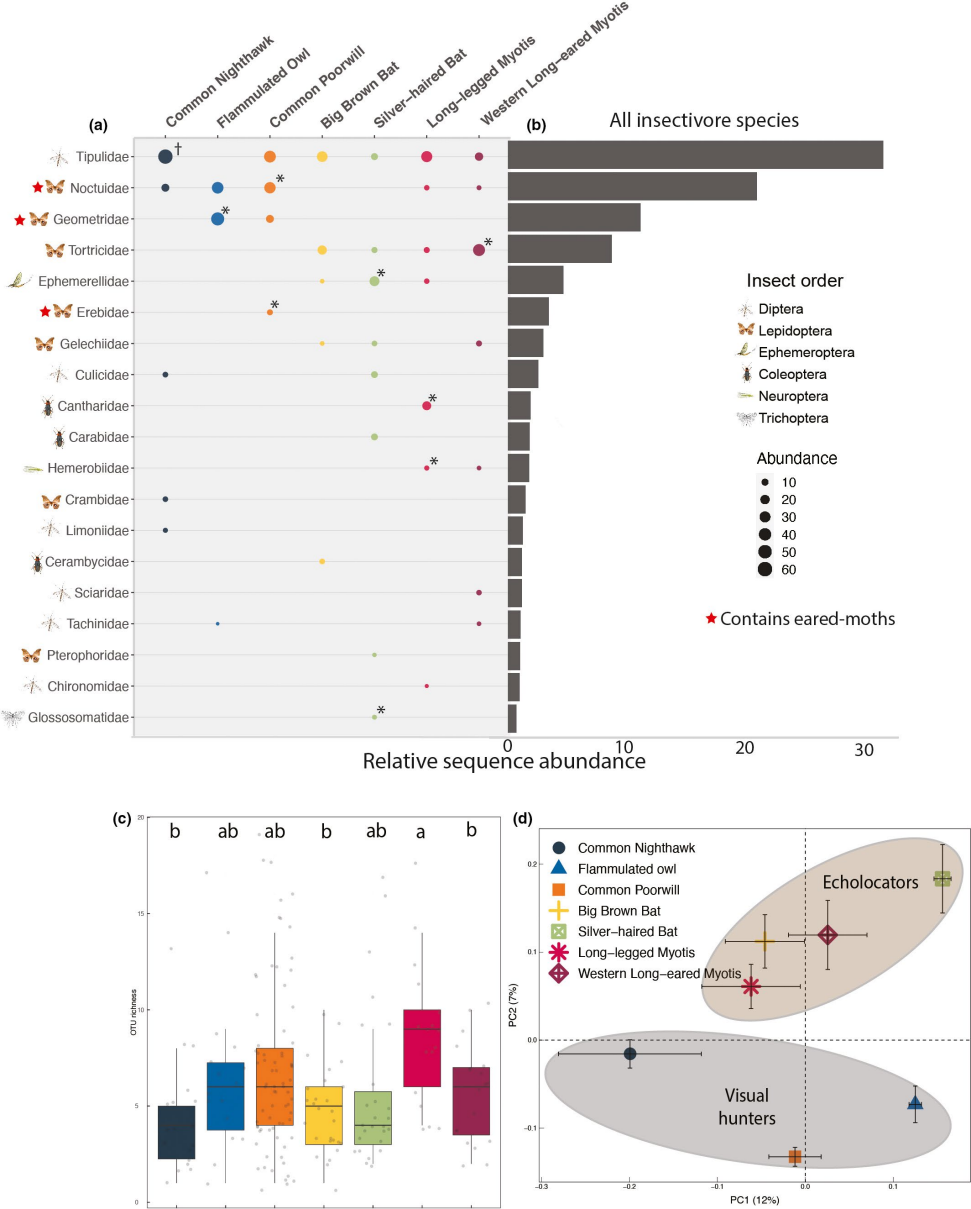
Variation in composition and richness of insectivore diets. (a) The percent relative sequence abundance of arthropod families found in the diets of seven nocturnal aerial insectivores. The size of points indicates the percent relative sequence abundance within each species and red outline indicates arthropod families significantly associated with the diet of an individual insectivore. Asterisks indicate the prey families maximally associated with each predator (^†^ *p* ≤ .07; *p ≤ .05) based on indicator species analyses (Table A3 in Appendix 1). The grey bars in (b) indicate the relative sequence abundance of insect families in the diets of all insectivores combined. Only insect families that represented >5.0% of any insectivore diet are shown. Diet richness (c) and principal coordinate analysis (PCoA = Multidimensional scaling) of diet composition of the seven sympatric nocturnal aerial insectivores (d) are based on OTUs of arthropod prey. Compositional means are represented by points, and error bars represent standard error for each insectivore species diet. Ellipses are overlayed to indicate variation between echolocating and visual predators

Moth families that contain species with ears (Miller & Surlykke, 2001) were maximally associated with the diets of NAIs that hunt visually (*p* < .01; Figure 1a; Table 2). The most abundant eared family, Noctuidae moths, occurred in the diets of 82% of Common Poorwills, 69% of Flammulated Owls, and 32% of Common Nighthawks sampled. Long‐legged Myotis fecal samples contained Noctuidae moths 35% of the time. However, just 16% of Long‐eared Myotis, 4% of Big Brown Bats, and no Silver‐haired Bats consumed Noctuid moths. Other eared moth families, including Geometridae, Sphingidae, and Erebidae, also occurred significantly more often in visual hunters’ diets, but rarely in bat diets (0%–15%).

**TABLE 2:** Indicator prey analysis results

Prey detection method					
Visual detection			Echolocation		
Insect family	Indicator value	*p*‐value	Insect family	Indicator value	*p*‐value
Erebidae*	0.51	<.001	Aphrophoridae	0.29	.002
Geometridae*	0.70	<.001	Cantharidae	0.24	.03
Noctuidae*	0.80	<.001	Carabidae	0.28	.005
Sphingidae*	0.29	.02	Cerambycidae	0.24	.03
			Chironimidae	0.29	.03
			Chrysopidae	0.29	.003
			Culicidae	0.37	.02
			Ephemerellidae	0.42	.002
			Gelechiidae	0.52	<.001
			Hemerobiidae	0.51	<.001
			Tortricidae	0.68	<.001

Note

Insect families significantly associated with each prey detection method (*p* < .05) using the “multipatt” function and 9999 permutations. *p*‐values were corrected for multiple comparisons. Insect families containing eared moths are indicated with an asterisk.

Conversely, the noneared moth family Tortricidae (mostly spruce budworm), was the most abundant family consumed more often by echolocators than by visual hunters (*p* < .001). We found Tortricidae in the diets of 65% of Long‐legged Myotis, 63% of Long‐Eared Myotis, 48% of Big Brown Bats, 23% of Silver‐haired Bats, but only 11% of Common Nighthawks, 5% of Common Poorwills, and 31% of Flammulated Owls. Ten other insect families were consumed more often by predators that use echolocation (Table 2, *p* < .02). Insect families that were recovered more often in diets of NAIs that hunt by aerial hawking (bats and nighthawks) partly corresponded with those associated with echolocation strategies (bats only, Table A2 in Appendix 1) with the exception of Limoniidae, Chironomidae, Carabidae, Chrysopidae, and Cerambycidae. This indicates that Common Nighthawks, as well as bat species, drove variation among these groups. All insect families and OTUs significantly associated with each NAI species’ diet can be found in Tables A3 and A4 in Appendix 1.

### 3.2 Dietary partitioning among species

In general, we saw low dietary overlap among species (Table 3) regardless of foraging behavior or prey detection method. The highest OTU overlap in diets occurred among bats, with the two smallest species, Long‐legged Myotis and Western Long‐eared Myotis overlapping the most (22%). Big Brown Bat diets overlapped slightly less with all other bat species (18%–21%). Among nocturnal birds, the highest overlap occurred between sit‐and‐wait predators, Common Poorwills, and Flammulated Owls (17%). Diet overlap between bats and birds was the lowest, with Flammulated Owls and Silver‐haired Bats overlapping by just 2%. However, Common Nighthawk diets overlapped similarly with all NAI species (10%–13%).

**TABLE 3:** Diet overlap among co‐occurring insectivores

	Common Nighthawks	Flammulated Owls	Common Poorwills	Big Brown Bats	Silver‐haired Bats	Long‐legged Myotis
Common Nighthawks	1					
Flammulated owls	13%	1				
Common Poorwills	11%	17%	1			
Big Brown Bats	13%	5%	5%	1		
Silver‐haired Bats	10%	2%	5%	19%	1	
Long‐legged Myotis	11%	8%	6%	21%	12%	1
Western Long‐eared Myotis	13%	8%	6%	18%	12%	22%

Note

Overlap in diet is based on the proportion of OTUs common to each species pair.

Controlling for differences between years, perMANOVA analyses on presence/absence data indicated that prey detection method (*R* ^2^ = .11, *p* = .001), foraging behavior (*R* ^2^ = .03, *p* = .001), species identity (*R* ^2^ = .09, *p* = .001), collection month (*R* ^2^ = .14, *p* = .001), plant community (*R* ^2^ = .09 *p* = .001), and the presence of water (*R* ^2^ = .01 *p* = .001) were all significant predictors of NAI diet (Table A5 in Appendix 1). The interaction between species and month also influenced diet composition (*R* ^2^ = .08, *p* = .001), highlighting the importance of seasonal variation within each NAI diet. Additional significant interactions occurred between species and plant community (*R* ^2^ = .02, *p* = .001), detection method and month (*R* ^2^ = .08, *p* = .001), detection method and plant community (*R* ^2^ = .01, *p* = .001), and month and plant community (*R* ^2^ = .03, *p* = .001). The total variation among insectivore diets explained by all significant main effects and interactions was 70%. We also performed perMANOVA analyses on Bray‐Curtis distances of compositional data. Relationships were similar, with prey detection method (*R* ^2^ = .04, *p* = .001), foraging behavior (*R* ^2^ = .02, *p* = .001), species identity (*R* ^2^ = .05, *p* = .001), collection month (*R* ^2^ = .08, *p* = .001), plant community (*R* ^2^ = .05 *p* = .001), and presence of water (*R* ^2^ = .01 *p* = .002) influencing variation in diets (Table A5 in Appendix 1). Overall, the model using compositional data explained 47% of the total variation among NAI diets. Pairwise comparisons indicated that the diets of all NAIs differed from each other (*p* < .006), except for Big Brown Bats and Long‐eared Myotis and, and Long‐legged Myotis and Long‐eared Myotis which had more similar diets (Figure 1d; Table A6 in Appendix 1). Big Brown Bats and Long‐legged Myotis only had marginally different diets (*p* = .07).

**FIGURE 2:**
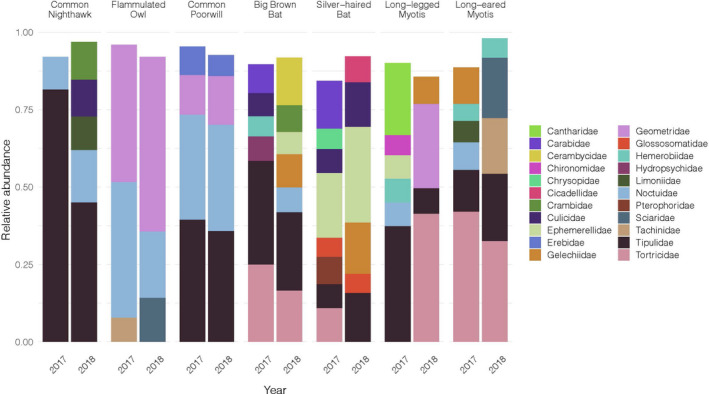
Diet variation between sampling years. Comparison of the relative abundances of insect families consumed by co‐occurring nocturnal aerial insectivores over a 2‐year period. Only families represented by >5.0% of total relative sequence abundance for an insectivore are shown

At the order level, Common Nighthawks and Long‐legged Myotis consumed mostly Diptera (true flies, 75% and 43% of diets, respectively). Common Poorwills and Flammulated Owls consumed mostly Lepidoptera (moths/butterflies; 63% and 88%, respectively). Silver‐haired bats were the only NAI to mostly consume Ephemeroptera (mayflies, 45%). In comparison, Big Brown Bats and Western Long‐eared Myotis consumed similar abundances of Lepidoptera (30% and 44%, respectively), and Diptera (31% and 43%, respectively). The top insect orders consumed by all NAI species combined were Lepidoptera followed by Diptera, Ephemeroptera, and Coleoptera (Figure 1b). Except for Long‐legged Myotis, the top insect families consumed for each NAI were consistent between 2017 and 2018 (Figure 2).

We found craneflies belonging to the genus *Tipula* in 35% of samples overall, more frequently than any other insect genera recovered. The most abundant and common prey OTU matched 100% with the family Tipulidae (BOLD: ADC2461, crane flies, Table 4). Morphological examination of specimens associated with this BIN confirmed it as *Tipula (Lunatipula) splendens* Doane 1901 (personal communication, Dr. Jon K. Gelhaus, 26 June 2021). This OTU occurred in 26% of all samples, in all NAI species diets except for Flammulated Owls, and was one of the most frequent *and* abundant prey items consumed by Common Poorwills, Common Nighthawks, Big Brown Bats, Long‐eared Myotis, and Long‐legged Myotis (Table 3).

**TABLE 4:** Top prey consumed by each insectivore species

Insectivore	BOLD accession	Highest taxonomic match	Order	Family	Frequency	Relative abundance
Common Nighthawks	ADC2461	Tipulidae	Diptera	Tipulidae	52%	31%
	ABA8386	*Tipula*	Diptera	Tipulidae	42%	6%
	AEB9328	Limoniidae	Diptera	Limoniidae	16%	14%
	AAF9002	*Tipula*	Diptera	Tipulidae	16%	1%
	ADQ3756	*Tipula*	Diptera	Tipulidae	16%	<1%
	AAA2144	*Xestia c‐nigrum*	Lepidoptera	Noctuidae	11%	5%
	ACU9148	*Limnophila*	Diptera	Limoniidae	11%	5%
Flammulated Owls	AAC0812	*Nepytia freemani*	Lepidoptera	Geometridae	31%	11%
	AAA4550	*Melanolophia imitata*	Lepidoptera	Geometridae	19%	11%
	AAA2632	*Noctua pronuba*	Lepidoptera	Noctuidae	19%	10%
	AAA6633	*Pero sp*.	Lepidoptera	Geometridae	19%	4%
	AAC6018	*Phaeoura mexicanaria*	Lepidoptera	Geometridae	13%	10%
	NA	*Mesogona sp*	Lepidoptera	Noctuidae	13%	5%
	ACF3238	*Euxoa satis*	Lepidoptera	Noctuidae	13%	2%
Common Poorwills	AAF9002	*Tipula*	Diptera	Tipulidae	32%	12%
	ADC2461	Tipulidae	Diptera	Tipulidae	25%	12%
	NA	Noctuidae	Lepidoptera	Noctuidae	25%	5%
	ABA8386	*Tipula*	Diptera	Tipulidae	25%	3%
	ABZ6253	*Grammia apantesis*	Lepidoptera	Erebidae	21%	4%
	AAA2632	*Noctua pronuba*	Lepidoptera	Noctuidae	21%	4%
	AAF0758	*Orthosia pulchella*	Lepidoptera	Noctuidae	19%	1%
	ACF3347	*Noctuidae Abagrotis sp*.	Lepidoptera	Noctuidae	17%	2%
Big Brown Bats	ABX5883	*Choristoneura freemani*	Lepidoptera	Tortricidae	37%	17%
	ADC2461	Tipulidae	Diptera	Tipulidae	33%	16%
	NA	Tipulidae	Diptera	Tipulidae	22%	4%
	NA	Aphrophoridae	Hemiptera	Aphrophoridae	22%	1%
	NA	Diptera	Diptera	NA	19%	3%
	ADQ3756	*Tipula*	Diptera	Tipulidae	15%	<1%
	AAC6388	Megasemum	Coleoptera	Cerambycidae	7%	6%
Silver‐haired Bats	AAV4027	*Ephemeroptera*	Ephemeroptera	NA	31%	11%
	NA	Culicidae	Diptera	Culicidae	19%	5%
	AAA2297	*Bryotropha similis*	Lepidoptera	Gelechiidae	19%	1%
	NA	*Amara*	Coleoptera	Carabidae	15%	6%
	ADQ9734	*Culicidae Aedes*	Diptera	Culicidae	15%	1%
	AAA1513	*Plutella xylostella*	Lepidoptera	Plutellidae	15%	1%
	AAZ1958	*Ephemerella dorothea*	Ephemeroptera	Ephemerellidae	8%	4%
Long‐legged Myotis	ADC2461	Tipulidae	Diptera	Tipulidae	47%	19%
	AAG0897	*Hemerobius conjunctus*	Neuroptera	Hemerobidae	41%	5%
	ABX5883	*Choristoneura freemani*	Lepidoptera	Tortricidae	35%	5%
	AAH0929	*Dichelotarsus excursus*	Coleoptera	Cantharidae	29%	17%
	AAA3570	*Coleotechnites*	Lepidoptera	Gelechiidae	24%	3%
	NA	Diptera	Diptera	NA	24%	1%
	NA	*Meleoma*	Neuroptera	Hemerobidae	24%	<1%
Western long‐eared Myotis	ABX5883	*Choristoneura freemani*	Lepidoptera	Tortricidae	47%	23%
	ADC2461	Tipulidae	Diptera	Tipulidae	26%	10%
	AAG0897	*Hemerobius conjunctus*	Neuroptera	Hemerobidae	26%	4%
	AAA3570	*Coleotechnites*	Lepidoptera	Gelechiidae	21%	6%
	AEB0463	Diptera	Diptera	NA	16%	12%
	AAH3943	Sciaridae	Diptera	Sciaridae	11%	5%
	NA	Ephemeroptera	Ephemeroptera	NA	11%	4%

Note

Taxonomic identification of the most frequent and abundant insect OTUs consumed by each nocturnal aerial insectivore.

The second most abundant OTU matched locally to *Choristoneura freemani* (western spruce budworm; BOLD: ABX5883) and was detected in 16% of all NAI samples. It was one of the top two OTUs consumed by most bat species but occurred in just one Common Nighthawk sample and two Common Poorwill samples.

### 3.3 Dietary breadth and turnover

From the fecal samples of all seven NAI species, we identified 73 arthropod families, 165 genera, and 382 OTUs. Silver‐haired bats had the widest diet breadth at the order (10) and family (36) levels (Table A7 in Appendix 1), whereas Common Poorwills consumed the greatest number of insect genera (75) and putative species or OTUs (154). We detected the fewest total OTUs in Common Nighthawk samples (50). Flammulated Owls had the highest variation or turnover among samples, whereas Common Poorwills, Common Nighthawks, and Silver‐haired bats had the lowest (Table 1). Long‐legged Myotis had the most OTU‐rich diet on average (Figure 1c), consuming more prey OTUs than Common Nighthawks, Big Brown Bats, and Long‐eared Myotis (*p* < .001; Table A8 in Appendix 1). NAI species, collection month, year, and plant community were significant predictors of dietary richness. Species identity had the greatest influence.

## 4 DISCUSSION

### 4.1 Eared moths are eaten more often by nocturnal birds than bats

In this study, we observed previously unreported dietary partitioning among co‐occurring nocturnal aerial insectivorous birds and bats. Variation in NAI diets corresponded with prey detection method based on both presence/absence and compositional data. This trend was supported primarily by Flammulated Owls, Common Poorwills, and to a lesser extent, Common Nighthawks successfully preying on eared moths more often than bats. Previous studies of the diets of these four bat species predominately used visual examinations of feces, which inhibited investigations of moth's auditory abilities and often resulted in order level taxonomic designations of prey. As such, this may be the first evidence that multiple families of eared moths largely avoid predation by a suite of bat species—relative to predation by sympatric nocturnal birds—in their natural environment.

Eared moths can detect bat echolocation calls from farther away than bats can detect moths, approximately ten times farther in the case of noctuid moths (Surlykke et al., 1999). As a result, moth adaptations to avoid bats (Hofstede & Ratcliffe, 2016; Waters, 2003) leave open niche space for nocturnal insectivorous birds that hunt visually. Complementary to visual detection methods, both Common Poorwills and Common Nighthawks have a velvety coating on wing and tail feathers adapted for quiet flight (Clark et al., 2020), which may make them difficult for eared moths to detect. Indeed, eared moths, especially noctuid moths, made up a large portion of Common Poorwill and Common Nighthawk diets, demonstrating the success of quiet flight adaptations.

Flammulated Owls also fly quietly and possess relatively long wings that allow them to move quickly (though perhaps without much agility) throughout the forest canopy (Johnson, 1997). Rather than aerial hawking, Flammulated Owls, like Common Poorwills, primarily use a sit‐and‐wait hunting strategy. This consists of flying from a perch inside the tree crown to capture insects resting in other areas of the same crown or adjacent trees (Reynolds & Linkhart, 1987). Together, these results indicate that birds that can ambush prey, rather than alert them with echolocation calls, can initiate successful attacks on eared insects at closer ranges.

The lower occurrence of eared moths in bat diets demonstrates the effectiveness of moth adaptations to bat predation (Hofstede & Ratcliffe, 2016). Still, Long‐legged and Long‐eared Myotis tended to consume eared moths at higher rates than the other bats in this study. Long‐legged Myotis makes echolocation calls at higher frequencies and detects prey at greater distances than Big Brown Bats and other myotis species, which may give it an advantage (Fenton & Bell, 1979; Saunders & Barclay, 1992). Alternatively, Long‐eared Myotis uses passive hearing and low‐amplitude calls while gleaning, which are undetectable by some eared moths (Faure et al., 1990). Gleaning by Myotis species evolved subsequent to echolocation strategies (Morales et al., 2019) and may be a counteradaptation to reduce detection by eared prey (Razak, 2018). However, gleaning may also have evolved as a general adaptation to hunting in cluttered areas (Brinkløv et al., 2010). An obvious counterstrategy to eared prey would be for bats to use a sit‐and‐wait hunting strategy. However, the physiology of most bats precludes them from leaping into flight (Schutt et al., 1997).

In addition to moths, ultrasonic hearing via tympanal organs has evolved independently within at least eight other insect orders, including Orthoptera, Mantodea, Blattodea, Hemiptera, Hymenoptera, Coleoptera, Neuroptera, and Diptera (Göpfert & Hennig, 2016; Hoy & Robert, 1996). Besides serving to detect and avoid predators, insect hearing has also evolved as a means of communication (Hoy & Robert, 1996). In Neuroptera, green lacewings can detect ultrasonic frequencies and avoid predation by bats (Miller, 1975), and a recent study indicates a similar ability in Myrmeleontidae of the Neuroptera (antlions) (Holderied et al., 2018). However, no insect family with known tympanal hearing abilities was significantly associated with bat diets in this study. Other insect families have evolved different mechanisms of hearing (e.g., Culicidae; Hoy & Robert, 1996), however these insects did not appear to avoid detection by bats more than birds.

### 4.2 Noneared prey partitioning among bats and birds

Though these results show a clear link between the ultrasonic hearing of moths and their higher occurrence in bird diets compared with bats, the partitioning of noneared insects is less clear. Moths in the family Tortricidae lack hearing organs (Fullard & Napoleone, 2001). This may explain why bats consumed Tortricidae in such high amounts and more often than nocturnal birds. The most commonly consumed Tortricidae moths, spruce budworms, tend to fly near treetops (Soutar & Fullard, 2004). Bat species in this study are known to forage in or near the forest canopy (Faure & Barclay, 1994; Menzel et al., 2005). Common Nighthawks that hunt high above the ground and Flammulated Owls that hawk from tree perches would also still encounter spruce budworm. Indeed, 11% of nighthawks and 31% of Flammulated Owls consumed Tortricidae in this study. However, for Common Poorwills that generally hunt only up to three meters above ground (Brigham & Barclay, 1992), spruce budworm may often be out of range. This would explain why Common Poorwills preyed on Tortricidae moths less often than all the other NAIs.

Common Nighthawks, on the other hand, shared similar diet overlap between both bats and birds. Their adaptations for silent flight may allow them to prey on Noctuidae and other eared moths, similar to Common Poorwills and Flammulated Owls. However, more like the bats in this study, Common Nighthawk diets were dominated by Diptera (75%). Common Nighthawks also consumed high proportions of Limoniidae (Table 4; Table A4 in Appendix 1), and Culicidae (mosquito family; Figure 1a) which may be more available to aerial hawkers that can forage over water bodies, than to sit‐and‐wait predators. Indeed, Culicidae was not found in the diet of any Common Poorwill or Flammulated Owl in our study. Previous investigations found that Common Poorwills only consumed prey >5 mm in length, despite a higher abundance of smaller insects in the environment, potentially due to visual constraints (Bayne & Brigham, 1995). We did not find any evidence contradicting this. However, since we used DNA instead of morphology to identify prey, we were unable to definitively determine prey size in many cases.

Previous studies suggest that variation in echolocation calls leads sympatric bat species to detect different prey resources, enabling coexistence (Razgour et al., 2011). However, such diet partitioning has not been shown empirically among the assemblage of bats in our study. Although overall diet composition did not differ or only marginally differed among Big Brown Bats, Long‐legged Myotis and Long‐eared Myotis (perMANOVA), we observed low overlap in the insect taxa consumed (18%–22%), suggesting some specialization. This pattern indicates that although these bats consume high abundances of the same species (i.e., spruce budworm), coexistence may be promoted due to differences in species consumed at lower frequencies. This hypothesis was also supported by stronger differences among species when analyzing presence/absence data compared with relative abundances, which is less sensitive to rare species. Additionally, minor differences in foraging locations may enable coexistence among sympatric species with similar foraging behaviors (Kent & Sherry, 2020), or resources like spruce budworm may be abundant enough to render partitioning unnecessary. Indeed, dietary partitioning may become more apparent when resources become more scarce (De León et al., 2014). Greater sampling efforts over longer periods of time and varying levels of resource availability in the future may reveal finer‐scale diet partitioning that we were unable to detect here.

Lepidoptera and Diptera dominated the diets of both bats and birds in this study. This may be partly due to bias associated with the primers used, which can underestimate other prey such as Coleoptera, Ephemeroptera, and Hymenoptera (Aldasoro et al., 2019). However, Alberdi et al. (2020) also observed Lepidoptera and Diptera dominating the diets of seven different bat species in Europe using the same primers as in this study. In that study, the use of additional primer pairs further confirmed their results, indicating that primer bias was not an issue. Nevertheless, preferential amplification of Lepidoptera and Diptera may inhibit observations of diet partitioning within or among other prey taxa such as Coleoptera and Ephemeroptera.

### 4.3 Conservation implications

North American avifauna have decreased in abundance by approximately 29% since 1970 (Rosenberg et al., 2019). Aerial insectivores are even more threatened (Nebel et al., 2010; Spiller & Dettmers, 2019). Bats face conservation threats globally and regionally (Frick et al., 2020). Though many factors contribute to declining population trends, decreases or changes in food availability play a role, making identification of key food sources important (Rosenberg et al., 2019; Spiller & Dettmers, 2019). The 73 arthropod families, 165 genera, and 382 OTUs identified in NAI diets in our study far exceed previous documentation, particularly at high taxonomic resolution, for most NAI species. However, there is still much work to be done with resolving the different taxa in the NAI diets. Tipulidae (crane flies) especially, were often not resolved beyond the family level here, yet were the most common order found in the diet of four of the seven NAIs.

Crane flies constitute the majority of prey for various wildlife, including snails, salamanders, and other Arthropoda (Lunghi et al., 2020), in addition to the species observed here (Table 4). A recent study found that crane fly abundance was a key predictor of the persistence of multiple sympatric bird species, and explained 39% of observed bird abundance (Carroll et al., 2015). This suggests that any decline in crane fly populations may be paired with future declines in avian populations. Monitoring crane fly populations may help identify high conservation priority areas as these insects are susceptible to plant community degradation and loss (Yadamsuren et al., 2015) and changes in water quality (Morse et al., 1994). Crane fly larvae in particular, are susceptible to desiccation (Pritchard, 1983), and prolonged drought or extreme heat caused by ongoing climate change may harm crane fly populations (Carroll et al., 2011). The importance of crane flies in NAI diets highlights the need for expanded analyses on crane fly ecology and conservation, especially as many species have yet to be described (Marshall, 2012).

**FIGURE A3:**
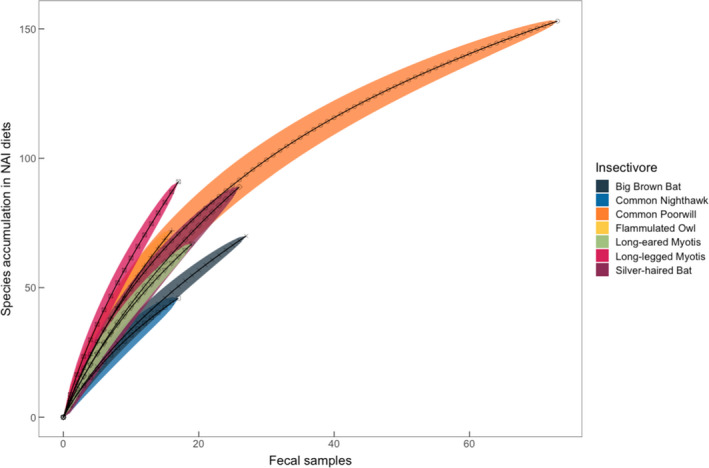
Sampling effort curve. Sampling effort curve of the number of unique arthropod OTUs detected in nocturnal insectivore diets per fecal sample

**FIGURE A4:**
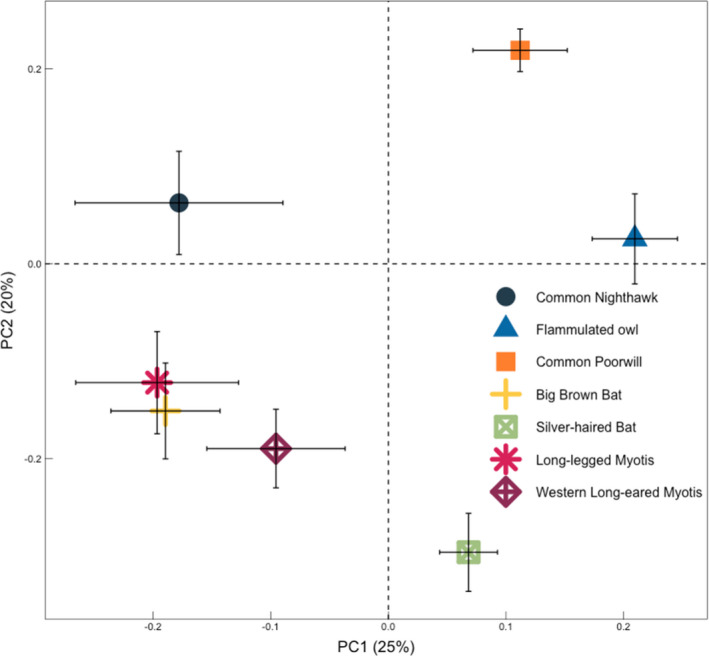
Principal coordinate analysis on presence/absence diet data. Principal coordinate analysis on Raup‐Crick distances of presence absence data from the diet of seven nocturnal aerial insectivores

Knowledge of NAI diets can also identify regulators of unwanted pests such as western spruce budworm, cutworm moths, and Douglas fir tussock moths that cause crop and forest damage. Western spruce budworm in particular, is a common conifer defoliator that reduces tree growth in the Pacific Northwest (Fierravanti et al., 2019). Because NAIs consume pests like spruce budworm in high and variable proportions, future research into the possible cascading effects on forest biomass and soil carbon retention may have global implications (Schmitz et al., 2017). Overall, our findings indicate that the evolutionary interactions between bats and moths may promote the coexistence of multi‐phyla predator communities. Future management practices that promote both eared and noneared prey insects may add stability to already threatened insectivore populations (Figures A3, A4.

## CONFLICT OF INTEREST

None declared.

## AUTHOR CONTRIBUTIONS

**Lorinda S. Bullington:** Data curation (lead); Formal analysis (lead); Investigation (lead); Methodology (equal); Validation (lead); Visualization (lead); Writing‐original draft (lead); Writing‐review & editing (lead). **Mathew T. Seidensticker:** Conceptualization (equal); Data curation (equal); Project administration (equal); Validation (equal). **Nathan Schwab:** Conceptualization (supporting); Methodology (equal); Writing‐review & editing (supporting). **Philip W. Ramsey:** Conceptualization (equal); Data curation (equal); Methodology (equal); Project administration (supporting); Writing‐original draft (supporting); Writing‐review & editing (supporting). **Kate Stone:** Conceptualization (lead); Data curation (supporting); Methodology (supporting); Project administration (equal); Supervision (equal); Writing‐original draft (supporting); Writing‐review & editing (supporting).

## Figures and Tables

**TABLE A4:**
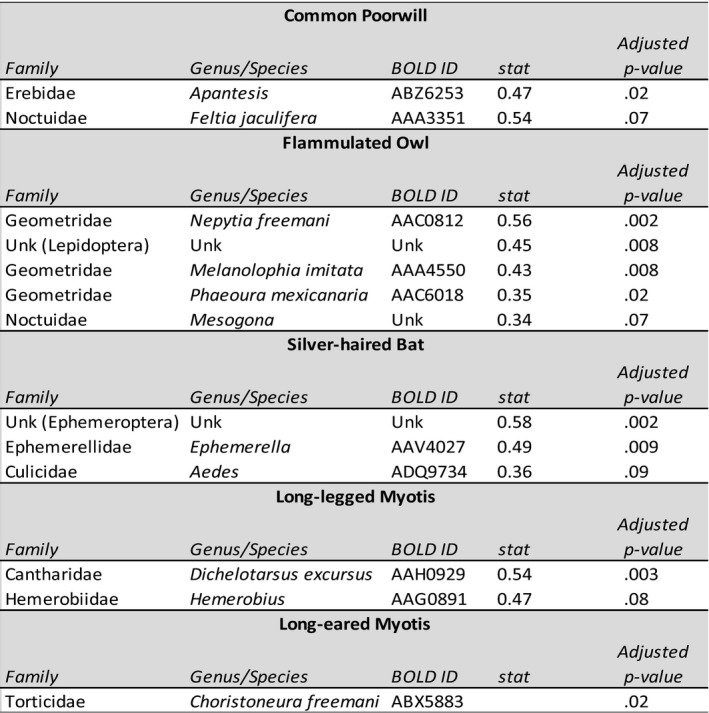
OTU level indicator prey analysis for each NAI species

*Note:* Insect OTUs significantly associated with each species using the “multipatt” function and 9999 permutations. *p*‐values were corrected for multiple comparisons. Insect families containing eared moths are indicated with an asterisk.

## Data Availability

All barcode sequences are publicly available on BOLD under the datasets MPG and MPGR. The full diet species table, metabarcode sequences, and taxonomy assignments can be found on Figshare, https://doi.org/10.6084/m9.figshare.16915159.

## APPENDIX 1

**TABLE A1:** Additional characteristics of seven sympatric nocturnal aerial insectivores

	Months sampled	Foraging style	Foraging range	Foraging timing
Common Nighthawks	June–Sept	Open aerial forager at a range of heights	Up to 400 Ha based on telemetry data	Crepuscular, nocturnal, occasionally diurnal
Flammulated Owls	May–Sept	Sit‐and‐wait (Gleans and flycatches from arboreal perch)	0.5 ha based on telemetry data	Nocturnal
Common Poorwills	May–Sept	Sit‐and‐wait (Sallys from ground or low perch; stays close to ground)	1–2 ha based on telemetry data	Crepuscular, Nocturnal
Big Brown Bats	May–Sept	Open aerial forager using strong, direct flight; 6–10 m above ground	1–2 km on average, up to 4.4 km (MT heritage project)	Crepuscular to nocturnal
Silver‐haired Bats	June–Aug	Open aerial forager using slow and maneuverable flight; tree tops and over open water	1–2 km on average, up to 3.4 km (MT Heritage project)	Crepuscular to nocturnal
Long‐legged Myotis	May–Sept	Open aerial forager using direct flight and long‐distance pursuit through and around forest canopies	up to 4 km from roosts or 448–647 Ha, but up to 3029 Ha (MT heritage project)	Nocturnal
Western Long‐eared Myotis	May–Aug	Open aerial forager and gleaner on leaves and bark in dense vegetation	1–2 ha, or less than 1 km (MT heritage project)	Crepuscular to nocturnal

**TABLE A2:** Family level indicator prey analysis for different foraging behaviors

Foraging behavior					
Sit‐and‐wait			Aerial hawking		
Insect family	Indicator value	Adjusted *p*‐value	Insect family	Indicator value	Adjusted *p*‐value
Noctuidae	0.84	<.001	Aphrophoridae	0.29	.02
Geometridae	0.75	<.001	Chrysopidae	0.29	.02
Erebidae	0.56	<.001	Culicidae	0.32	.002
Sphingidae	0.32	.003	Ephemerellidae	0.45	<.001
			Gelechiidae	0.48	.007
			Hemerobiidae	0.46	<.001
			Limoniidae	0.32	.02
			Tortricidae	0.63	<.001

Note

Insect families significantly associated with each foraging behavior using the “multipatt” function and 9999 permutations. *p*‐values were corrected for multiple comparisons. Insect families containing eared moths are indicated with an asterisk.

**TABLE A3:** Family level indicator prey analysis for each NAI species

Family	Stat	Adjusted *p*‐value
Common Nighthawk		
Tipulidae	0.43	.07
Common Poorwill		
Erebidae	0.61	<.001
Noctuidae	0.54	.005
Flammulated Owl		
Geometridae	0.76	<.001
Silver‐haired Bat		
Plutellidae	0.37	.03
Ephemerelliidae	0.42	.03
Cicadellidae	0.34	.05
Glossosomatidae	0.34	.05
Long‐legged Myotis		
Cantharidae	0.54	<.001
Hemerobiidae	0.49	.01
Long‐eared Myotis		
Torticidae	0.56	.006

Note

Insect families significantly associated with each species using the “multipatt” function and 9999 permutations. *p*‐values were corrected for multiple comparisons. Insect families containing eared moths are indicated with an asterisk.

**TABLE A5:** Results from perMANOVA analysis

Bray‐Curtis (relative read abundance)	*R*‐squared	*p*‐value	Presence/absence (Raup‐Crick)	*R*‐squared	*p*‐value
Detection method	.04	.001	Detection method	.12	.001
Foraging behavior	.02	.001	Foraging behavior	.03	.001
Species	.05	.001	Species	.09	.001
Collection month	.08	.001	Collection month	.14	.001
Plant community	.05	.001	Plant community	.09	.001
Water body present	.01	.002	Water body present	.01	.001
Detection: Plant community	.01	.001	Detection: Plant community	.01	.001
Foraging behavior: Plant community	.01	.002	Foraging behavior: Plant community	.01	.02
Species: Month	.07	.001	Species: Month	.08	.001
Species: Plant community	.02	.001	Species: Plant community	.02	.001
Detection: Month	.05	.001	Detection: Month	.08	.001
Month: Plant community	.03	.001	Month: Plant community	.03	.001

Note

Results from perMANOVA analysis of nocturnal aerial insectivore diets over a two‐year period, with permutations constrained within years. Analysis was performed on Raup‐Crick transformed presence/absence data and Hellinger transformed Bray‐Curtis distances of rarefied sequences.

**TABLE A6:** Results from pairwise perMANOVA analyses

Species comparison	Bray‐Curtis (relative read abundance)		Presence/absence data	
	*R*‐squared	Adjusted *p*‐value	*R*‐squared	Adjusted *p*‐value
Common Nighthawks: Common Poorwills	.07	.001	.07	.001
Common Nighthawks: Big Brown Bat	.09	.005	.09	.006
Common Nighthawks: Flammulated Owls	.19	.001	.19	.001
Common Nighthawks: Silver‐haired Bats	.17	.001	.17	.001
Common Nighthawks: Long‐eared Myotis	.12	.001	.12	.001
Common Nighthawks: Long‐legged Myotis	.13	.007	.13	.006
Common Poorwill: Big Brown Bat	.14	.001	.14	.001
Common Poorwill: Flammulated Owl	.07	.001	.07	.001
Common Poorwill: Silver‐haired Bat	.19	.001	.19	.001
Common Poorwill: Long‐legged Myotis	.12	.001	.12	.001
Common Poorwill: Long‐eared Myotis	.13	.001	.13	.001
Big Brown Bat: Flammulated Owl	.18	.001	.18	.001
Big Brown Bat: Silver‐haired Bat	.14	.001	.14	.001
Big Brown Bat: Long‐eared Myotis	.03	.29	.03	.26
Big Brown Bat: Long‐legged Myotis	.05	.11	.05	.073
Flammulated Owl: Silver‐haired Bat	.2	.001	.2	.001
Flammulated Owl: Long‐eared Myotis	.16	.001	.16	.002
Flammulated Owl: Long‐legged Myotis	.23	.001	.23	.001
Silver‐haired Bat: Long‐eared Myotis	.14	.001	.13	.001
Silver‐haired Bat: Long‐legged Myotis	.19	.001	.19	.001
Long‐eared Myotis: Long‐legged Myotis	.04	.35	.04	.37

Note

*p*‐values are adjusted for multiple comparisons using the Benjamini and Hochberg method.

**TABLE A7:** Dietary niche breadth

	Orders	Families	Genera	OTUs
Common Nighthawks	4	13	21	50
Flammulated Owls	7	17	39	72
Common Poorwills	8	21	75	154
Big Brown Bats	7	26	26	70
Silver‐haired Bats	10	36	45	89
Long‐legged Myotis	7	30	40	91
Western long‐eared Myotis	9	29	30	67

Note

Summary of unique prey taxa counts from DNA barcoding analysis of fecal contents of seven nocturnal aerial insectivores.

**TABLE A8:** Summary of diversity analyses

Predictor	Likelihood ratio	*p*‐value
Richness (Poisson)		
Species	190	.**001**
Habitat	185	.07
Month	181	.**001**
Year	180	.**001**
Habitat: Month	168	.**001**
Shannon's diversity (Gaussian)		
Habitat: Month	40.6	.**001**

Note

Results from generalized linear models (GLM) using either a Poisson or Gaussian distribution to test for differences in diet richness and diversity of seven sympatric nocturnal aerial insectivores.

Bold values indicate significant predictors (*p* = .001).
